# Estimated plasma volume status as a prognostic indicator in myocardial infarction and heart failure: insights from the MIMIC-IV database

**DOI:** 10.3389/fcvm.2025.1499378

**Published:** 2025-03-13

**Authors:** Bin Luo, Zheng Ma, Guoyong Zhang, Xue Jiang, Caixia Guo

**Affiliations:** Cardiovascular Center, Beijing Tongren Hospital, Capital Medical University, Beijing, China

**Keywords:** estimated plasma volume status, myocardial infarction, heart failure, prognosis, risk stratification

## Abstract

**Background:**

Myocardial infarction (MI) complicated by heart failure (HF) is a common and severe clinical condition associated with poor outcomes. Estimated plasma volume status (ePVS), a marker of congestion derived from hemoglobin and hematocrit, has shown promise in predicting outcomes in various cardiovascular diseases. This study aimed to investigate the relationship between ePVS and both short-term and long-term prognosis in patients with MI complicated by HF.

**Methods:**

A retrospective cohort study was conducted using data from the Medical Information Mart for Intensive Care IV (MIMIC-IV) database, including 3,238 patients with MI complicated by HF. Patients were stratified into quartiles based on ePVS values. The primary outcomes were in-hospital mortality, 180-day mortality, and 1-year mortality. Kaplan–Meier curves, multivariate Cox regression analysis, and subgroup analyses were performed to assess the relationship between ePVS and outcomes.

**Results:**

Kaplan–Meier analysis showed significant differences in survival rates across ePVS quartiles for all outcomes (*P* < 0.001). Multivariate logistic regression analysis revealed that patients in the highest quartile of ePVS (Q4 vs. Q1) had an independently increased risk of in-hospital mortality (OR 1.58, 95% CI 1.16–2.13, *P* = 0.003). Cox regression analysis further demonstrated that higher ePVS (Q4 vs. Q1) was associated with an increased risk of 180-day mortality (HR 1.45, 95% CI 1.19–1.75, *P* < 0.001) and 1-year mortality (HR 1.51, 95% CI 1.27–1.80, *P* < 0.001). Both Kaplan–Meier survival curves and restricted cubic spline models confirmed a positive association between ePVS and long-term mortality risks.The association between ePVS and long-term outcomes was stronger than for in-hospital mortality. Subgroup analyses revealed that the relationship between ePVS and long-term mortality was more pronounced in patients with systolic blood pressure below 140 mmHg, lower LODS and OASIS scores, and those without hemorrhagic disorders or anemia (*P* for interaction <0.05).

**Conclusion:**

ePVS was an independent predictor of both short-term and long-term mortality in patients with MI complicated by HF. Its prognostic value was particularly significant for long-term outcomes, suggesting its potential utility in risk stratification and guiding treatment strategies for this high-risk population.

## 1 Introduction

Myocardial infarction (MI) is a leading cause of heart failure (HF), with approximately 40% of MI patients developing HF either during the acute phase or during follow-up (1). Patients with in-hospital heart failure had a 13% higher in-hospital mortality rate and a 14% higher 30-day mortality rate compared to those without heart failure during their acute myocardial infarction hospitalization (2). This patient population consumes substantial healthcare resources, placing a significant burden on healthcare systems and society (3).

Fluid volume management is crucial for patients with MI complicated by HF, yet it presents considerable challenges (4). These patients often experience fluid overload, while excessive diuresis can lead to renal function deterioration and electrolyte imbalances (5). Therefore, accurate assessment of volume status is essential for guiding treatment. Plasma volume (PV) has been recognized as a potential prognostic indicator in this population (6). However, direct measurement of PV is impractical in clinical settings. Consequently, estimated plasma volume status (ePVS), derived from hemoglobin and hematocrit values, has emerged as a promising prognostic indicator in various cardiovascular conditions (6).

In recent years, several studies have demonstrated the prognostic value of ePVS in various cardiovascular disease populations. In HF patients, Duarte et al. found that higher ePVS was associated with poorer outcomes in both HFrEF and HFpEF patients (7). For acute MI patients, Chen et al. showed that higher ePVS was correlated with increased in-hospital mortality (8). Kawai et al. further confirmed that ePVS could predict not only in-hospital mortality but also long-term mortality in AMI patients (9).

Despite the established prognostic value of ePVS in MI and HF separately, research specifically examining ePVS in patients with MI complicated by HF remains scarce. This high-risk population warrants dedicated investigation due to its unique pathophysiological characteristics. Our study addressed this knowledge gap by evaluating the predictive value of ePVS for both short-term and long-term outcomes in patients with MI complicated by HF. The findings of this investigation might provide novel insights for enhancing risk stratification and developing personalized treatment strategies in this complex patient cohort. By elucidating the role of ePVS in this specific context, our study aimed to contribute to improved patient management protocols and potentially better clinical outcomes.

## 2 Methods

### 2.1 Participants and study design

This retrospective study utilized data from the Medical Information Mart for Intensive Care IV (MIMIC-IV) database, a publicly accessible repository containing comprehensive clinical information on patients admitted to Beth Israel Deaconess Medical Center (BIDMC) between 2008 and 2019 (10). The MIMIC-IV database encompasses a wide range of patient data, including length of stay, laboratory test results, medication regimens, vital signs, and other pertinent clinical information. To ensure patient confidentiality, all personal identifiers were replaced with randomly generated codes, obviating the need for individual informed consent or ethical approval.

**Figure 1:**
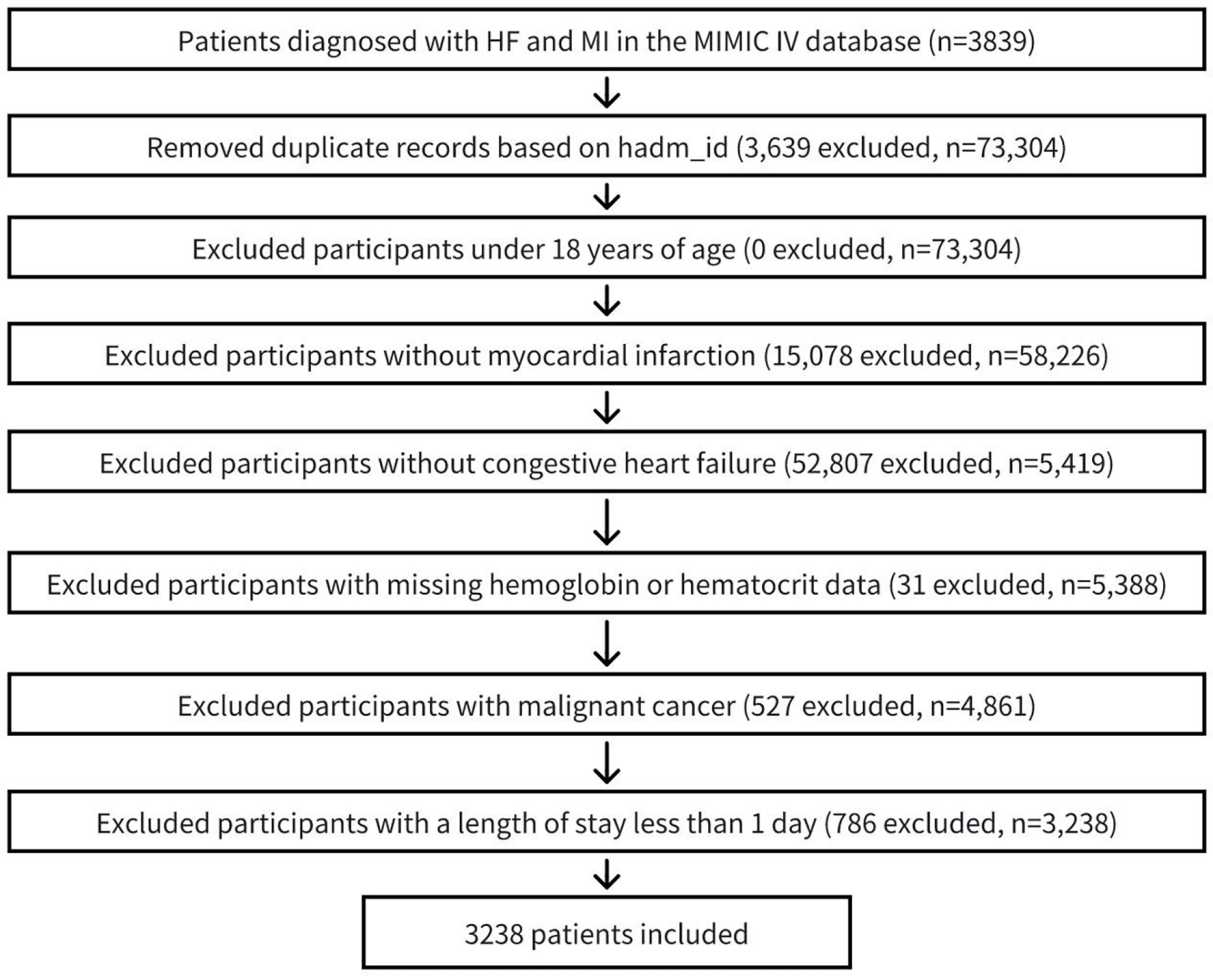
Flow chart of patient selection process. MI, myocardial infarction; CHF, congestive heart failure.

The study population comprised patients diagnosed with myocardial infarction (MI) and congestive heart failure (CHF), identified using International Classification of Diseases, 9th and 10th Revision codes. Only the first hospital admission for each eligible patient was included in the analysis. Exclusion criteria were as follows: (1) age below 18 years, (2) absence of MI and CHF diagnoses, (3) insufficient hemoglobin or hematocrit data for calculating ePVS, and (4) hospital stay shorter than 24 h (5)with malignant cancer. Following the application of these criteria, a final cohort of 3,238 patients was selected for analysis. The patient selection process was detailed in Figure 1.

### 2.2 Data collection

Structured Query Language (SQL) was applied to extract the relevant medical information from the MIMIC-IV database. The following data was obtained: (1) demographics, including age, sex and race. (2) vital signs, including body mass index (BMI), pulse oximetry derived oxygen saturation (spo2), systolic blood pressure (SBP) and diastolic blood pressure (DBP). (3) laboratory indicators, including white blood cell (WBC), red blood cell (RBC), prothrombin time (PT), platelet, hemoglobin, hematocrit, creatinine, blood urea nitrogen (BUN), glucose, sodium and potassium. (4) clinical score, including logistic organ dysfunction system (LODS) score, Oxford Acute Severity of Illness Score (OASIS), Simplified Acute Physiology Score (SAPSII) and Sequential Organ Failure Assessment Score (SOFA). (5) Cardiac-related variables, including left ventricular ejection fraction(LVEF), ST-elevation myocardial infarction(STEMI), non-ST-elevation myocardial infarction(NSTEMI), percutaneous coronary intervention(PCI). (6) comorbidities, including sepsis, acute kidney injury (AKI), chronic kidney disease (CKD), chronic obstructive pulmonary disease (COPD), atrial fibrillation (AF), cardiomyopathy, stroke, cerebrovascular disease, diabetes, hypertension, hemorrhagic disorders, anemia and usage of diuretics. (7) outcomes, including in-hospital death, 180-day death and 1-year death, which were all defined as all-cause mortality.

### 2.3 Grouping and outcome

The Duarte formula and Hakim formula are commonly used methods to calculate ePVS (7).

The Strauss-derived Duarte formula calculates ePVS based on hematocrit and hemoglobin levels (6, 11), as follows:

ePVS(mL/g)=100times(1-Hematocrit)/Hemoglobin(g/dL)

The Hakim formula, which estimates plasma volume from hematocrit and dry body weight (12). The equations for actual and ideal plasma volumes are as follows:

ActualPlasmaVolume=(1-Hematocrit)×(a+btimesBodyWeight(kg))

IdealPlasmaVolume=ctimesBodyWeight(kg)

Where the constants a, b, and c differ by sex:

• For males: a = 1,530, b = 41.0, c = 39
• For females: a = 864, b = 47.9, c = 40

Finally, ePVS was calculated as follows:

$$\begin{array}{rl} \mathrm{ePVS}\;(\mathrm{mL}/g)\;= & [(\mathrm{Actual}\;\mathrm{Plasma}\;\mathrm{Volume}\text{-}\mathrm{Ideal}\;\mathrm{Plasma}\;\mathrm{Volume})/\\ & \mathrm{Ideal}\;\mathrm{Plasma}\;\mathrm{Volume}]\;\;\times \;100 \end{array}$$

Participants were divided according to ePVS quartiles based on Duarte formula, all patients were divided into four groups: Q1 (ePVS <5.02, *n* = 811), Q2(5.02 <ePVS <6.13, *n* = 808), Q2(6.13 <ePVS <7.61, *n* = 811), Q4 (ePVS >7.61, *n* = 808). The short-term endpoint of the study was in-hospital death. Long-term outcomes focused on 180-day death and 1-year death.

### 2.4 Statistical analysis

Continuous variables were expressed as mean ± standard deviation (SD) or median and interquartile range (IQR) based on the distribution of data. The Kolmogorov–Smirnov test was used to verify the normality of the data. Data with a normal distribution were analyzed using ANOVA analysis; for data with a skewed distribution, the Kruskal–Wallis H test was used. All categorical data were expressed as frequency (percentile). Chi-square test or Fisher test were applied to compare the categorical variables. Multivariate analyses were performed to assess the associations between ePVS and clinical outcomes. Missing values were handled using median imputation. Logistic regression was used to evaluate in-hospital mortality, while Cox proportional hazards models were employed to analyze 180-day and 1-year mortality. Results are presented as odds ratios (OR) for in-hospital mortality and hazard ratios (HR) for 180-day and 1-year mortality, both with corresponding 95% confidence intervals (CI). Model 1 was unadjusted, while Model 2 accounted for sex, age, and race. Model 3 was further adjusted for additional parameters, including SBP, Spo2, hypertension, stroke, glucose, WBC, and platelet. The covariates included in Model 3 were selected based on clinical experience and stepwise regression, with only those variables showing a significance level of *p* < 0.05 being retained. Generalized additive model (GAM) and smoothed curve fitting were utilized to elucidate the potential non-linear relationship between ePVS and in-hospital mortality. Restricted cubic spline (RCS) curves were employed to explore the association between ePVS and both 180-day and 1-year mortality. Kaplan–Meier survival analysis was employed to assess the incidence rate of secondary endpoints (180-day and 1-year death) for each group, and their differences were assessed through log-rank tests. Subgroup analysis was conducted to estimate the consistency of the effect in different groups including age (≤65, >65years), sex, BMI (≤30, >30 kg/m2), SBP (≤140, >140 mmHg), LODS score (low, middle, high), OASIS score (low, middle, high), SOFA score (≤9, >9 and ≤12, >12) and SAPSII score (low, middle, high).

Statistical significance was defined as a two-tailed *p*-value of less than 0.05. All statistical analyses were performed using the R software environment (Version 4.3.2; The R Foundation; available at http://www.R-project.org).

## 3 Results

### 3.1 Baseline characteristics of the participants

The final analysis included 3,238 participants, comprising 2,005 males (61.92%) and 1,233 females (38.08%). The mean age of the study population was 72.09 years (SD ± 12.23). In-hospital mortality was observed in 449 patients (13.87%). Follow-up data revealed that 948 patients (29.28%) died within 180 days of admission, while 1-year mortality reached 1,127 patients (34.81%).

The baseline characteristics were shown in Table 1. Participants in higher ePVS groups were more likely to be older and have higher levels of Spo2, creatinine, BUN, LODS score, OASIS score, SOFA score, SAPSII score, sepsis, AKI, CKD, COPD, AF, stroke, cerebrovascular disease and diabetes, but lower levels of male proportion, SBP, DBP, WBC, RBC, hemoglobin, hematocrit, glucose, sodium, cardiomyopathy and hypertension than those in lower ePVS groups (all *P* < 0.05). Furthermore, in higher quartiles of ePVS groups, notably higher rates of in-hospital death, 180-day death and 1-year death were observed (*P* < 0.001). There was no statistically significant difference in other indicators among different groups (all *p* value >0.05).

**Table 1:** Baseline characteristics of patients with HF and MI grouped by quartiles of ePVS.

Characteristics	Total	Quartiles of ePVS				*P*-value
		Q1	Q2	Q3	Q4	
		ePVS <5.02	5.02 < ePVS <6.13	6.13 <ePVS <7.61	ePVS >7.61	
*N*	3,238	811	808	811	808	
Age (year), mean ± SD	72.09 ± 12.23	69.14 ± 12.51	73.13 ± 12.07	73.81 ± 11.73	72.30 ± 12.09	<0.001
Sex, *n* (%)						<0.001
Male	2,005 (61.92%)	627 (77.31%)	460 (56.93%)	462 (56.97%)	456 (56.44%)	
Female	1,233 (38.08%)	184 (22.69%)	348 (43.07%)	349 (43.03%)	352 (43.56%)	
Race, *n* (%)						0.001
White	2,258 (69.73%)	538 (66.34%)	574 (71.04%)	586 (72.26%)	560 (69.31%)	
Black/African American	219 (6.76%)	47 (5.80%)	49 (6.06%)	48 (5.92%)	75 (9.28%)	
Hispanic/Latino	79 (2.44%)	14 (1.73%)	23 (2.85%)	18 (2.22%)	24 (2.97%)	
Asian	74 (2.29%)	19 (2.34%)	22 (2.72%)	19 (2.34%)	14 (1.73%)	
Other	608 (18.78%)	193 (23.80%)	140 (17.33%)	140 (17.26%)	135 (16.71%)	
Vital sign						
BMI (kg/m^2^), median (IQR)	28.06 (24.24–32.58)	28.65 (25.14–32.61)	28.06 (23.98–32.81)	27.71 (24.11–32.32)	27.65 (23.97–32.57)	0.119
Spo2 (%), mean ± SD	96.70 ± 3.87	96.36 ± 3.95	96.45 ± 4.04	96.94 ± 3.64	97.05 ± 3.81	<0.001
SBP (mmHg), median (IQR)	119.00 (104.00–135.00)	120.00 (104.00–135.00)	120.00 (104.75–136.25)	119.00 (105.00–135.00)	115.00 (102.00–133.25)	0.039
DBP (mmHg), median (IQR)	63.00 (53.00–76.00)	69.00 (58.00–81.00)	63.00 (54.00–74.00)	61.00 (53.00–73.00)	60.00 (50.00–71.00)	<0.001
Laboratory						
WBC (×10^9^/L), median (IQR)	10.00 (7.60–13.90)	10.70 (7.90–14.70)	10.10 (7.60–14.10)	9.80 (7.50–13.55)	9.75 (7.30–13.40)	<0.001
RBC (×10^12^/L), median (IQR)	3.65 (3.14–4.18)	4.53 (4.23–4.87)	3.86 (3.67–4.07)	3.38 (3.22–3.58)	2.82 (2.60–3.06)	<0.001
Platelet (×10^9^/L), median (IQR)	200.00 (153.25–259.00)	198.00 (161.00–249.00)	204.50 (157.75–263.25)	205.00 (156.50–261.50)	189.00 (137.00–264.00)	0.006
Hemoglobin (g/dl), median (IQR)	10.96 ± 2.22	13.85 ± 1.19	11.63 ± 0.47	10.14 ± 0.47	8.20 ± 0.86	<0.001
Hematocrit (%), median (IQR)	33.50 ± 6.46	41.83 ± 3.71	35.41 ± 1.71	31.09 ± 1.62	25.64 ± 2.69	<0.001
Creatinine (mg/dl), median (IQR)	1.75 ± 1.58	1.32 ± 0.75	1.52 ± 1.25	1.90 ± 1.66	2.27 ± 2.13	<0.001
BUN (mg/dl), median (IQR)	27.00 (18.00–43.00)	21.00 (17.00–30.00)	25.50 (18.00–39.00)	30.00 (19.00–45.00)	35.00 (22.00–56.00)	<0.001
Glucose (mg/dl), median (IQR)	156.64 ± 82.50	166.39 ± 86.13	157.99 ± 78.70	150.92 ± 78.77	151.27 ± 85.29	<0.001
Sodium (mmol/L), median (IQR)	138.34 ± 4.68	138.75 ± 4.50	138.43 ± 4.49	138.35 ± 4.72	137.85 ± 4.97	0.006
Potassium (mmol/L), median (IQR)	4.32 ± 0.72	4.32 ± 0.69	4.27 ± 0.73	4.28 ± 0.69	4.40 ± 0.75	<0.001
Clinical score						
LODS score, mean ± SD	5.00 (3.00–7.00)	4.00 (2.00–7.00)	5.00 (3.00–7.00)	6.00 (3.00–8.00)	6.00 (4.00–8.00)	<0.001
OASIS score, mean ± SD	33.00 (27.00–40.00)	32.00 (26.00–38.00)	33.00 (27.00–40.00)	33.00 (27.00–41.00)	33.00 (27.00–40.00)	<0.001
SAPSII score, mean ± SD	39.00 (31.00–48.00)	36.00 (28.00–45.00)	37.00 (31.00–46.25)	40.00 (33.00–48.50)	41.00 (33.00–49.00)	<0.001
SOFA score, mean ± SD	5.00 (3.00–8.00)	5.00 (3.00–8.00)	5.00 (3.00–8.00)	5.00 (3.00–8.00)	6.00 (4.00–9.00)	<0.001
LVEF(%)	40.00 (30.00–55.00)	40.00 (25.00–50.00)	40.00 (30.00–55.00)	40.00 (30.00–55.00)	45.00 (35.00–55.00)	<0.001
MI subtypes						<0.001
STEMI	734 (22.67%)	247 (30.46%)	175 (21.66%)	140 (17.26%)	172 (21.29%)	
NSTEMI	573 (17.70%)	124 (15.29%)	133 (16.46%)	146 (18.00%)	170 (21.04%)	
Unclear	1,931 (59.64%)	440 (54.25%)	500 (61.88%)	525 (64.73%)	466 (57.67%)	
PCI	501 (15.47%)	176 (21.70%)	130 (16.09%)	105 (12.95%)	90 (11.14%)	<0.001
Comorbidities, *n* (%)						
Sepsis	1,788 (55.22%)	393 (48.46%)	447 (55.32%)	476 (58.69%)	472 (58.42%)	<0.001
AKI	2,570 (79.37%)	617 (76.08%)	641 (79.33%)	660 (81.38%)	652 (80.69%)	0.041
CKD	1,323 (40.86%)	229 (28.24%)	269 (33.29%)	378 (46.61%)	447 (55.32%)	<0.001
Chronic pulmonary disease	1,087 (33.57%)	236 (29.10%)	291 (36.01%)	276 (34.03%)	284 (35.15%)	0.015
Atrial fibrillation	1,745 (53.89%)	416 (51.29%)	402 (49.75%)	436 (53.76%)	491 (60.77%)	<0.001
Cardiomyopathy	463 (14.30%)	138 (17.02%)	107 (13.24%)	92 (11.34%)	126 (15.59%)	0.006
Stroke	591 (18.25%)	121 (14.92%)	162 (20.05%)	143 (17.63%)	165 (20.42%)	0.014
Cerebrovascular disease	503 (15.53%)	105 (12.95%)	133 (16.46%)	152 (18.74%)	113 (13.99%)	0.006
Diabetes	1,534 (47.37%)	312 (38.47%)	377 (46.66%)	401 (49.45%)	444 (54.95%)	<0.001
Hypertension	958 (29.59%)	260 (32.06%)	287 (35.52%)	244 (30.09%)	167 (20.67%)	<0.001
Hemorrhagic disorders	1,313 (40.55%)	292 (36.00%)	283 (35.02%)	299 (36.87%)	439 (54.33%)	<0.001
Anemia	1,516 (46.82%)	220 (27.13%)	295 (36.51%)	429 (52.90%)	572 (70.79%)	<0.001
Diuretic treatment	2,539 (78.41%)	621 (76.57%)	633 (78.34%)	640 (78.91%)	645 (79.83%)	0.440
Outcomes, *n* (%)						
In-hospital death	449 (13.87%)	93 (11.47%)	109 (13.49%)	111 (13.69%)	136 (16.83%)	0.019
180-day Death	948 (29.28%)	183 (22.56%)	225 (27.85%)	260 (32.06%)	280 (34.65%)	<0.001
1-year Death	1,127 (34.81%)	217 (26.76%)	267 (33.04%)	301 (37.11%)	342 (42.33%)	<0.001

IQR, interquartile range; SD, standard deviation; BMI, body mass index; SBP, systolic blood pressure; DBP, diastolic blood pressure; WBC, white blood cell count; RDW, red cell distribution width; MCH, mean corpuscular hemoglobin; MCHC, mean corpuscular hemoglobin concentration; MCV, mean corpuscular volume; BUN, blood urea nitrogen; ALT, alanine aminotransferase; AST, aspartate aminotransferase; LDH, lactate dehydrogenase; ALP, alkaline phosphatase; INR, international normalized ratio; PT, prothrombin time; PTT, partial thromboplastin time; CRP, C-reactive protein; MELD, model for end-stage liver disease; LODS, logistic organ dysfunction system; OASIS, oxford acute severity of illness score; SAPSII, simplified acute physiology score II; SOFA, sequential organ failure assessment; GCS, glasgow coma scale; SIRS, systemic inflammatory response syndrome; AKI, acute kidney injury; CKD, chronic kidney disease.

### 3.2 Correlation of the ePVS with in-hospital death

**Figure 2:**
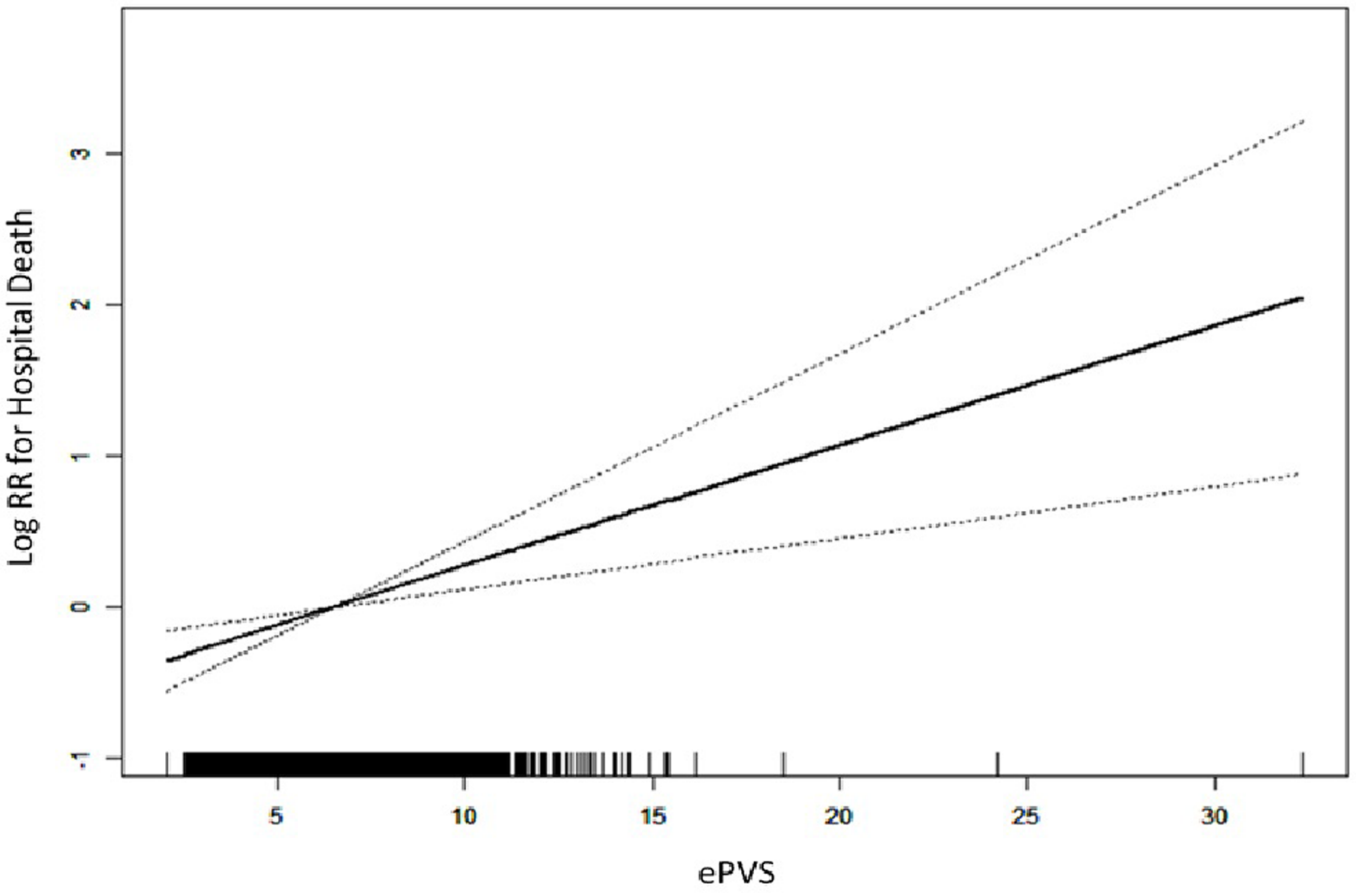
Generalized additive model for in-hospital mortality prediction.

Logistic analysis model revealed the associations of ePVS with in-hospital death (Table 2). In the initial unadjusted Model 1, a significant positive association was observed between ePVS and in-hospital death [Q4 vs. Q1: OR (95% CI): 1.56 (1.18, 2.08), *p* = 0.002, *P* for trend = 0.003]. In Model 2, after adjusting for sex, age and race, ePVS still showed a positive correlation with in-hospital death [Q4 vs. Q1: OR (95% CI): 1.47 (1.10, 1.97), *p* = 0.010, *P* for trend = 0.011]. In the fully adjusted Model 3, the ePVS was still independently related to the increased risk of in-hospital death [Q4 vs. Q1: OR (95% CI): 1.58 (1.16, 2.13), *p* = 0.003, *P* for trend = 0.003]. Furthermore, when ePVS was considered as a continuous variable in the model for analysis, we observed that for each unit increase in the ePVS, the risk of in-hospital death increased approximately 8% in Model 1 (*p* = 0.001), 7% in Model 2 (*p* = 0.002), 8% in Model 3 (*p* = 0.002) respectively. Consistently, The GAM and smoothed curve fitting showed a positive linear relationship between ePVS and log risk ratio (RR) for in-hospital death (Figure 2). The smooth term fitting and Wald test for the smooth term showed a significant *p*-value (*p* < 0.001), indicating that the relationship between ePVS and in-hospital death is non-linear. The results related to the Hakim Formula are provided in the supplementary materials (Supplementary Table S2 and Figure S1).

**Table 2:** Associations of ePVS with in-hospital death (in logistic analysis model), 180-day death and 1-year death (in cox analysis model).

Model	In-hospital death			180-day death			1-year death		
	OR (95% CI)	*P* Value	*P* for trend	HR (95% CI)	*P* Value	*P* for trend	HR (95% CI)	*P* Value	*P* for trend
Model 1			0.003			<0.001			<0.001
Quartile 1	Reference			Reference			Reference		
Quartile 2	1.20 (0.90, 1.62)	0.219		1.28 (1.05, 1.55)	0.014		1.29 (1.08, 1.54)	0.006	
Quartile 3	1.22 (0.91, 1.64)	0.178		1.48 (1.22, 1.79)	<0.001		1.47 (1.23, 1.75)	<0.001	
Quartile 4	1.56 (1.18, 2.08)	0.002		1.62 (1.35, 1.95)	<0.001		1.71 (1.44, 2.02)	<0.001	
ePVS	1.08 (1.03, 1.13)	0.001		1.08 (1.05, 1.12)	<0.001		1.09 (1.06, 1.11)	<0.001	
Model 2			0.011			<0.001			<0.001
Quartile 1	Reference			Reference			Reference		
Quartile 2	1.10 (0.81, 1.49)	0.546		1.09 (0.89, 1.33)	0.409		1.09 (0.91, 1.31)	0.346	
Quartile 3	1.10 (0.81, 1.49)	0.540		1.24 (1.03, 1.51)	0.027		1.23 (1.03, 1.47)	0.024	
Quartile 4	1.47 (1.10, 1.97)	0.010		1.43 (1.18, 1.73)	<0.001		1.50 (1.26, 1.78)	<0.001	
ePVS	1.07 (1.03, 1.13)	0.002		1.07 (1.04, 1.10)	<0.001		1.07 (1.04, 1.10)	<0.001	
Model 3			0.003			<0.001			<0.001
Quartile 1	Reference			Reference			Reference		
Quartile 2	1.15 (0.84, 1.57)	0.389		1.10 (0.90, 1.34)	0.347		1.11 (0.92, 1.33)	0.280	
Quartile 3	1.21 (0.89, 1.66)	0.222		1.28 (1.05, 1.55)	0.013		1.26 (1.06, 1.51)	0.010	
Quartile 4	1.58 (1.16, 2.13)	0.003		1.45 (1.19, 1.75)	<0.001		1.51 (1.27, 1.80)	<0.001	
ePVS	1.08 (1.03, 1.13)	0.002		1.07 (1.04, 1.10)	<0.001		1.07 (1.04, 1.10)	<0.001	

Model 2 adjust for: sex, age, race. Model 3 adjust for: sex, age, race, SBP; Spo2; hypertension; stroke; glucose; WBC; platelet.

### 3.3 Correlation of the ePVS with 180-day death and 1-year death

Cox analysis model showed the associations of ePVS with 180-day death and 1-year death (Table 2). In the initial unadjusted Model 1, 180-day death and 1-year death risk were significantly higher in the high-ePVS group (180-day death Q4 vs. Q1: HR (95% CI): 1.62 (1.35, 1.95), *p* < 0.001, *P* for trend <0.001; 1-year death Q4 vs. Q1: HR (95% CI): 1.71 (1.44, 2.02), *p* < 0.001, *P* for trend <0.001).

**Figure 3:**
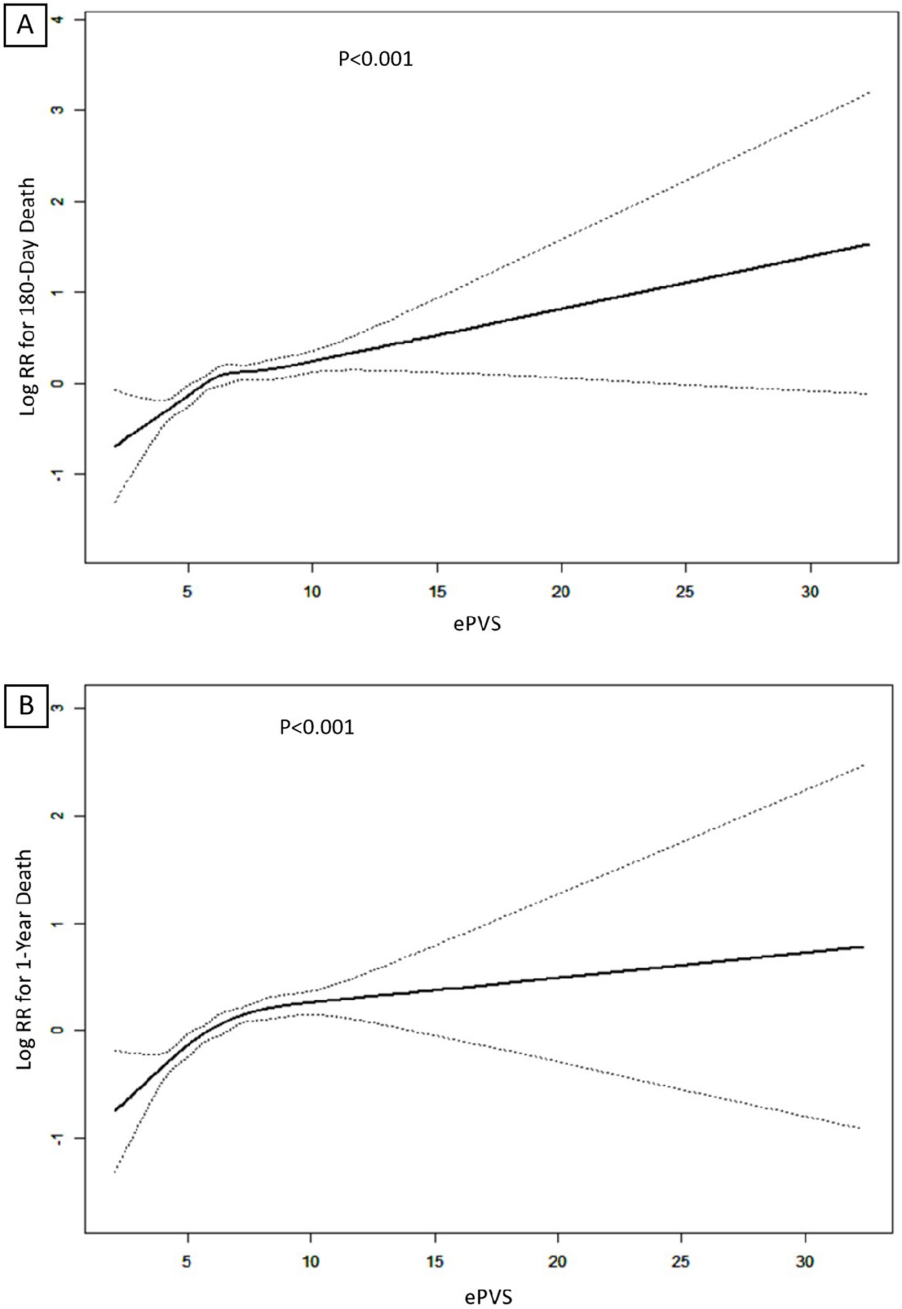
**(A)** Restricted cubic spline (RCS) plots of ePVS for predicting 180-day mortality; **(B)** RCS plots of ePVS for predicting 1-year mortality predictions.

**Figure 4:**
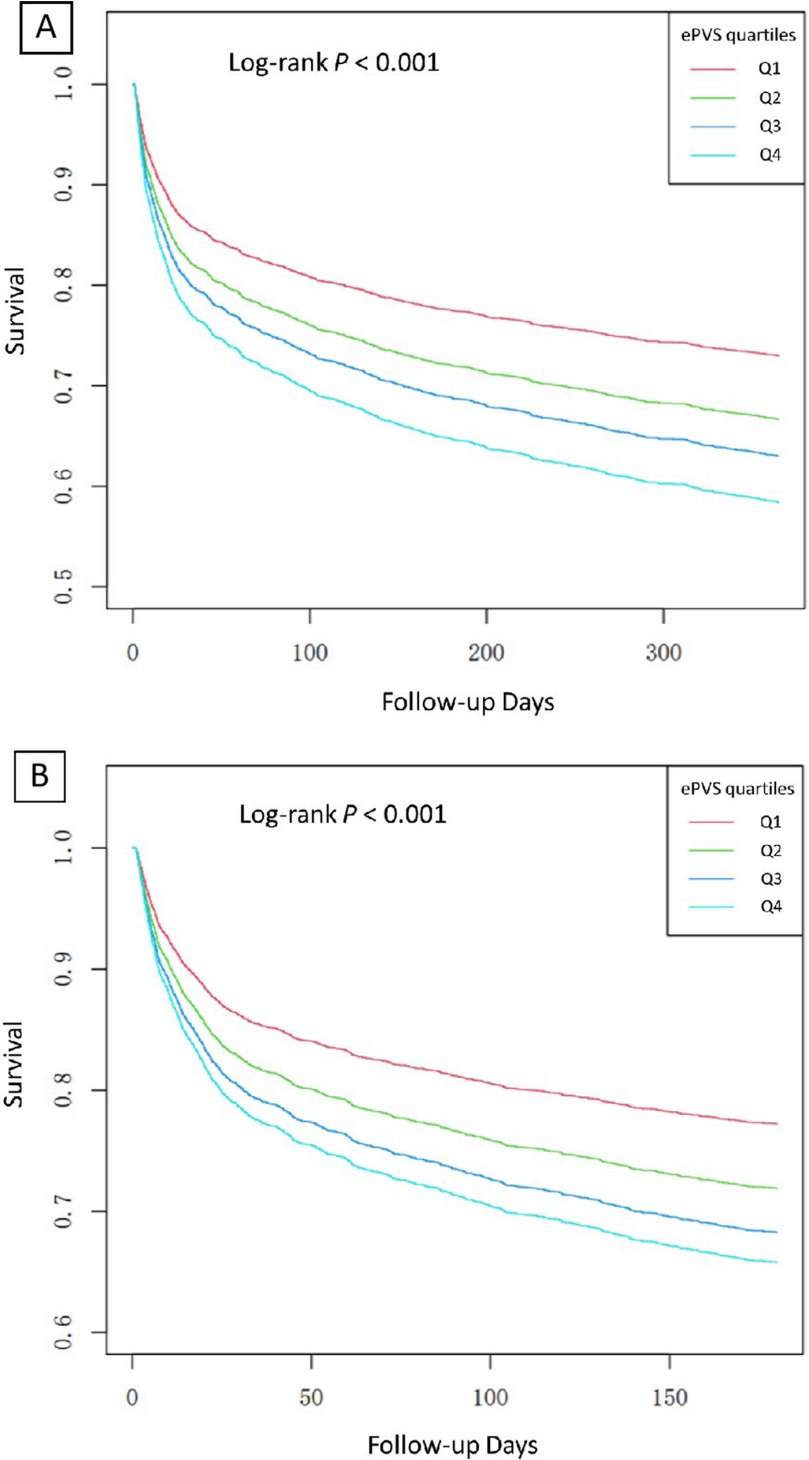
**(A)** Kaplan–Meier analysis of ePVS for predicting 180-day mortality; **(B)** Kaplan–Meier analysis of ePVS for predicting 1-year mortality predictions.

In Model 2, after adjusting for sex, age and race, ePVS still showed a positive correlation with secondary outcomes (180-day death Q4 vs. Q1: HR (95% CI): 1.43 (1.18, 1.73), *p* < 0.001, *P* for trend <0.001; 1-year death Q4 vs. Q1: HR (95% CI): 1.50 (1.26, 1.78), *p* < 0.001, *P* for trend <0.001). In the fully adjusted Model 3, the ePVS was still independently related to the increased risk of secondary outcomes (180-day death Q4 vs. Q1: HR (95% CI): 1.45 (1.19, 1.75), *p* < 0.001, *P* for trend <0.001; 1-year death Q4 vs. Q1: HR (95% CI): 1.51 (1.27, 1.80), *p* < 0.001, *P* for trend <0.001). When ePVS was considered as a continuous variable in the model for analysis, we observed that for each unit increase in the ePVS, the risk of 180-day death and 1-year death increased approximately 7% and 7% in Model 3 (*p* < 0.001) respectively. Subsequently, we modelled the RCS curves, which predicted a positive relationship between ePVS and log RR for 180-day death (Figure 3A) and 1-year death (Figure 3B). The likelihood ratio test and Wald test for the smooth term showed a significant *p*-value (all *p* < 0.001), indicating that the relationship between ePVS and 180-day death and 1-year death is non-linear. Kaplan–Meier survival analysis curves were plotted, which showed that patients with a higher ePVS had a significantly higher risk of 180-day (Figure 4A) and 1-year death (Figure 4B) than those with a lower ePVS (log-rank *P* < 0.001). The results related to the Hakim Formula are provided in the supplementary materials (Supplementary Table S2 and Figure S2–S5).

### 3.4 Subgroup analysis

In subgroup analysis, for in-hospital death, significant interactions were observed in OASIS score subgroups (*p* for interaction = 0.005), hemorrhagic disorders subgroups (*p* for interaction = 0.043) and anemia subgroups (*p* for interaction = 0.038) (Table 3). Among patients with low and middle OASIS score, higher ePVS was significantly associated with an increased risk of in-hospital death. Conversely, for those with high OASIS score, no significant correlation was found. For 180-day death, significant interactions were observed in SBP subgroups (*p* for interaction = 0.026), LODS score subgroups (*p* for interaction = 0.025), OASIS score subgroups (*p* for interaction <0.001), hemorrhagic disorders subgroups (*p* for interaction = 0.002) and anemia subgroups (*p* for interaction = 0.012). The ePVS seemed to have more prominence of its 180-day death predictive value in patients with SBP of 140 mmHg or below, low LODS score, low and middle OASIS score, and those without hemorrhagic disorders or anemia. Similar results were observed in 1-year death, in which significant interactions were observed in SBP (*p* for interaction = 0.021), LODS score (*p* for interaction = 0.002), OASIS score (*p* for interaction <0.001), hemorrhagic disorders subgroups (*p* for interaction = 0.010) and anemia subgroups (*p* for interaction = 0.010). The ePVS appeared to be a more effective predictor for 1-year death in patients with SBP of 140 mmHg or below, low LODS score, low and middle OASIS score, and those without hemorrhagic disorders or anemia. No significant interactions were observed in the other analyzed subgroups.

**Table 3:** Subgroup analysis of ePVS with in-hospital death (in logistic analysis model), 180-day death and 1-year death (in cox analysis model).

Subgroup	In-hospital death			180-day death			1-year death		
	OR(95%CI)	*P* value	P(interaction)	HR(95%CI)	*P* value	P(interaction)	HR(95%CI)	*P* value	*P* (interaction)
Age			0.089			0.484			0.776
≤65	1.01 (0.91,1.12)	0.870		1.01 (0.99, 1.12)	0.116		1.01 (0.99,1.12)	0.022	
>65	1.11 (1.05,1.17)	<0.001		1.11 (1.04, 1.11)	<0.001		1.11 (1.04,1.11)	<0.001	
Sex			0.287			0.128			0.332
Male	1.10 (1.04,1.17)	<0.001		1.10 (1.05, 1.13)	<0.001		1.10 (1.05,1.12)	<0.001	
Female	1.05 (0.96,1.14)	0.285		1.05 (0.98, 1.09)	0.173		1.05 (0.98,1.1)	0.035	
BMI			0.737			0.567			0.374
≤30	1.06 (0.99,1.14)	0.091		1.06 (1.01, 1.12)	0.016		1.06 (1.01,1.11)	0.018	
>30	1.09 (0.99,1.2)	0.097		1.09 (1.02, 1.16)	0.008		1.09 (1.02,1.16)	0.002	
SBP			0.236			0.026			0.021
≤140	1.09 (1.04,1.15)	<0.001		1.08 (1.05,1.12)	<0.001		1.09 (1.05,1.12)	<0.001	
>140	1.01 (0.88,1.15)	0.927		0.98 (0.90,1.07)	0.650		0.99 (0.92,1.07)	0.780	
LODS score			0.070			0.025			0.002
Low	1.27 (1.06,1.53)	0.009		1.27 (1.05, 1.25)	0.002		1.27 (1.05,1.26)	<0.001	
Middle	1.03 (0.92,1.14)	0.638		1.03 (0.98, 1.1)	0.168		1.03 (0.98,1.1)	0.074	
High	1.01 (0.95,1.07)	0.735		1.01 (0.96, 1.04)	0.896		1.01 (0.96,1.04)	0.982	
OASIS score			0.005			<0.001			<0.001
Low	1.30 (1.13,1.48)	<0.001		1.30 (1.15, 1.33)	<0.001		1.30 (1.15,1.28)	<0.001	
Middle	1.10 (1.02,1.2)	0.020		1.10 (1.03, 1.14)	0.004		1.10 (1.03,1.13)	0.002	
High	1.01 (0.95,1.08)	0.701		1.01 (0.95, 1.04)	0.835		1.01 (0.95,1.04)	0.922	
SOFA score			0.142			0.209			0.206
≤9	1.08 (1.01,1.15)	0.018		1.06 (1.03,1.1)	0.001		1.07 (1.03,1.10)	<0.001	
>9, ≤12	1.08 (0.98,1.19)	0.110		1.07 (1.00,1.14)	0.040		1.07 (1.00,1.13)	0.043	
>12	0.94 (0.82,1.07)	0.338		0.98 (0.90,1.07)	0.652		0.99 (0.91,1.07)	0.758	
SAPSII score			0.285			0.098			0.060
Low	1.18 (1.02,1.36)	0.028		1.18 (1.03, 1.2)	0.010		1.18 (1.03,1.2)	0.002	
Middle	1.04 (0.94,1.14)	0.469		1.04 (1.02, 1.13)	0.011		1.04 (1.02,1.12)	0.007	
High	1.04 (0.97,1.1)			1.04 (0.97, 1.06)	0.452		1.04 (0.97,1.06)	0.373	
LVEF			1.000			0.160			0.124
LVEF≥45%	1.1 (0.97, 1.25)	0.121		1.12 (1.05, 1.21)	0.001		1.14 (1.07, 1.21)	<0.001	
LVEF<45%	1.1 (0.96, 1.28)	0.176		1.04 (0.95, 1.14)	0.439		1.05 (0.97, 1.14)	0.226	
MI subtypes			0.618			0.485			0.215
STEMI	1.13 (1.04, 1.23)	0.006		1.12 (1.07, 1.18)	<0.001		1.13 (1.08, 1.19)	<0.001	
NSTEMI	1.09 (0.98, 1.21)	0.096		1.09 (1.02, 1.16)	0.010		1.08 (1.01, 1.14)	0.017	
PCI			0.689			0.155			0.053
Yes	1.1 (0.99, 1.23)	0.071		1.13 (1.05, 1.22)	0.002		1.14 (1.07, 1.22)	<0.001	
No	1.08 (1.02, 1.14)	0.005		1.06 (1.03, 1.09)	<0.001		1.06 (1.03, 1.09)	<0.001	
Hemorrhagic disorders			0.043			0.002			0.010
Yes	1.03 (0.96, 1.1)	0.392		1.03 (0.98, 1.07)	0.250		1.04 (1, 1.08)	0.061	
No	1.14 (1.06, 1.22)	<0.001		1.13 (1.08, 1.17)	<0.001		1.12 (1.07, 1.16)	<0.001	
Anemia			0.038			0.012			0.010
Yes	1.06 (0.98, 1.13)	0.128		1.05 (1.01, 1.09)	0.028		1.05 (1.01, 1.09)	0.017	
No	1.18 (1.09, 1.27)	<0.001		1.13 (1.09, 1.18)	<0.001		1.13 (1.08, 1.18)	<0.001	
Diuretic treatment			0.255			0.376			0.893
Yes	1.1 (1.05, 1.16)	<0.001		1.08 (1.04, 1.12)	<0.001		1.07 (1.04, 1.11)	<0.001	
No	1.03 (0.93, 1.14)	0.549		1.04 (0.98, 1.12)	0.215		1.08 (1.01, 1.15)	0.016	

Adjust for: sex, age, race, marriage, SBP; Spo2; hypertension; stroke; glucose; WBC; platelet; statin; aspirin.

## 4 Discussion

Our study aimed to investigate the relationship between ePVS and short-term and long-term outcomes in patients with myocardial infarction (MI) complicated by heart failure (HF). The results demonstrated that elevated ePVS burden was independently associated with increased mortality risk across multiple time points, including in-hospital, 180-day, and 1-year follow-up periods. Subgroup analyses revealed that this association between higher ePVS burden and increased mortality was particularly pronounced among patients with systolic blood pressure below 140 mmHg and those presenting with lower Logistic Organ Dysfunction Score (LODS) and Oxford Acute Severity of Illness Score (OASIS).These results suggested that ePVS was a valuable prognostic indicator for both short-term and long-term outcomes in patients with MI complicated by HF, potentially serving as a simple and effective tool for risk stratification in this high-risk population.

Several previous studies have investigated the prognostic value of ePVS in various cardiovascular conditions. Duarte et al. examined 5,002 patients with chronic heart failure and found that higher ePVS was associated with increased all-cause mortality (HR 1.06, 95% CI 1.03–1.08) over a median follow-up of 21 months (7). In a study of 1,175 acute myocardial infarction patients, Chen et al. demonstrated that elevated ePVS was correlated with higher in-hospital mortality (OR 1.31, 95% CI 1.12–1.53) (8). Kawai et al. further confirmed the prognostic value of ePVS in 1,280 AMI patients, showing that higher ePVS predicted both in-hospital mortality (OR 1.27, 95% CI 1.06–1.52) and long-term mortality (HR 1.20, 95% CI 1.07–1.34) over a median follow-up of 3.2 years (9). These studies consistently demonstrated that ePVS was a valuable prognostic indicator in various cardiovascular conditions. The pathophysiological basis for the association between elevated ePVS and poor outcomes in cardiovascular patients has been explored in several studies. Increased plasma volume could lead to congestion, which was associated with organ dysfunction and adverse outcomes in heart failure patients (13). Moreover, plasma volume expansion has been linked to neurohormonal activation, inflammation, and oxidative stress, all of which contribute to the progression of cardiovascular disease (14). In patients with myocardial infarction, elevated ePVS may reflect the severity of myocardial damage and subsequent neurohormonal activation, potentially explaining its association with poor outcomes (8).

While previous studies have established the prognostic value of ePVS in separate populations of heart failure and myocardial infarction patients, there was a lack of evidence specifically addressing the high-risk group of patients with myocardial infarction complicated by heart failure. This population represents a significant clinical challenge, with complex fluid management needs and a high risk of adverse outcomes (4).

Our study addressed this gap by focusing on this specific patient group, providing novel insights into the prognostic value of ePVS in this high—risk population. We demonstrated that ePVS was significantly associated with both short—term and long—term outcomes in patients with MI complicated by HF, with a stronger association observed for long—term outcomes. Clinically, this finding suggested that ePVS might be an essential marker for identifying patients at sustained risk, allowing clinicians to adopt more proactive, longer—term monitoring and interventions aimed at preventing adverse events (15). In contrast, short—term outcomes, while important, might reflect more immediate physiological responses, which could be mitigated with acute management strategies (16). We used the Duarte formula to calculate ePVS and obtained comparable predictive performance, with the related results provided in the supplementary materials. However, the Duarte formula involves a more complicated calculation process and requires gender-specific adjustments (12). In contrast, the Hakim formula is simpler and more straightforward while still demonstrating good predictive accuracy (6).

Interestingly, our subgroup analysis revealed that the association between higher ePVS and increased mortality was particularly pronounced among patients with SBP below 140 mmHg, those with lower LODS and OASIS scores and in patients without hemorrhagic disorders or anemia. This finding suggested that ePVS might be especially useful in identifying high—risk patients who might otherwise be considered lower risk based on traditional clinical parameters. The stronger association in patients with lower blood pressure could be attributed to the fact that these patients may have had less physiological reserve to compensate for volume overload, making them more susceptible to the adverse effects of increased plasma volume. The stronger association between ePVS and outcomes in patients without hemorrhagic disorders or anemia is particularly noteworthy. This finding likely reflects the fact that these conditions can directly affect hemoglobin and hematocrit values, which are used to calculate ePVS (17, 18). In patients with bleeding or anemia, ePVS calculations may not accurately reflect true plasma volume status, potentially reducing its prognostic utility in these populations (19, 20). Similarly, the enhanced prognostic value in patients with lower clinical severity scores suggested that ePVS might have captured additional risk information not reflected in these conventional scoring systems. These subgroup findings highlighted the potential of ePVS to refine risk stratification, particularly in patients who might have appeared less severely ill by other measures. The integration of ePVS into clinical practice could therefore lead to more personalized treatment strategies, potentially improving outcomes in this challenging patient population.

The relationship between elevated ePVS and increased mortality could be explained by several pathophysiological mechanisms. Acute MI led to cardiomyocyte death and subsequent left ventricular remodeling, which was exacerbated by volume overload. In a rat model of MI, increased plasma volume showed to activate stretch-sensitive signaling pathways in surviving cardiomyocytes, leading to hypertrophy and fibrosis (21). This maladaptive remodeling contributed to progressive cardiac dysfunction over time. Furthermore, volume overload in MI-induced HF activated the renin-angiotensin-aldosterone system (RAAS), as demonstrated in animal studies (22). Chronic RAAS activation promoted sodium retention, vasoconstriction, and further volume expansion, creating a vicious cycle that worsened heart failure over time. At the cellular level, increased plasma volume led to sustained myocardial stretch, which had been shown to induce pro-inflammatory cytokine expression in cardiomyocytes (23). This chronic inflammation contributed to ongoing myocardial damage and dysfunction. Additionally, volume overload in HF was associated with increased oxidative stress and mitochondrial dysfunction in cardiac tissue (24). These processes impaired cellular energy metabolism and contributed to progressive cardiac deterioration. The stronger association between ePVS and long-term mortality could be attributed to the cumulative effects of these pathological processes over time. While acute volume overload could be partially compensated for in the short term, chronic volume expansion led to sustained neurohormonal activation, ongoing inflammation, and progressive cardiac remodeling. This gradual deterioration explained why ePVS might be a more potent predictor of long-term outcomes. Moreover, elevated ePVS might reflect a more severe initial myocardial injury or a reduced capacity for compensatory mechanisms, both of which would manifest as poorer long-term prognosis in MI patients with HF. Although reduced ePVS values are theoretically associated with poor outcomes, our study did not find such a relationship. While decreased ePVS may reflect dehydration or hypovolemia in general populations, patients with MI complicated by heart failure typically experience volume overload (25), not depletion. Myocardial injury triggers neurohormonal activation, leading to fluid retention and plasma volume expansion (26). In these patients, lower ePVS values likely reflect effective management of congestion through fluid and diuretic therapy, rather than pathological volume loss.

This study had several limitations that should be acknowledged. Firstly, the retrospective design limited causal inference. Secondly, the single-center nature of the study might restrict the generalizability of our findings. Thirdly, the lack of long-term follow-up data beyond one year prevented assessment of the impact of ePVS on extended outcomes. Specific limitations included the absence of important clinical variables such as left ventricular ejection fraction and NT-proBNP levels, which could have provided additional insights into cardiac function and prognosis. The study population, derived from a single database, might not fully represent the diverse spectrum of MI patients with HF. ePVS was calculated only at admission, precluding analysis of its dynamic changes during hospitalization and recovery. Despite adjusting for multiple variables, residual confounding factors could not be completely ruled out. Economic constraints prevented repeated ePVS measurements, which could have provided valuable information on its temporal trends. Future research should address these limitations by conducting prospective, multi-center studies with longer follow-up periods, incorporating a more comprehensive set of clinical variables, and exploring the dynamic changes of ePVS throughout the course of MI and HF. Additionally, studies in diverse patient populations are needed to confirm the generalizability of our findings.

## 5 Conclusion

In conclusion, this study demonstrates the significant prognostic value of ePVS in patients with myocardial infarction complicated by heart failure. ePVS showed strong associations with both short-term and long-term mortality, particularly for 180-day and 1-year outcomes. The simplicity and cost-effectiveness of ePVS calculation make it a promising tool for risk stratification in various clinical settings. Future prospective, multi-center studies with longer follow-up periods are needed to validate these findings and explore ePVS-guided treatment strategies, potentially improving patient management and outcomes in this high-risk population.

## Data Availability

The datasets analyzed for this study can be found in the Medical Information Mart for Intensive Care (MIMIC-IV) database (https://mimic.mit.edu/).
