# Supplementary material for: Estimated plasma volume status as a prognostic indicator in myocardial infarction and heart failure: insights from the MIMIC-IV database
Source: Front Cardiovasc Med. 2025 Mar 13;12:1499378. doi: 10.3389/fcvm.2025.1499378 (PMC11947685; doi:10.3389/fcvm.2025.1499378)

# Supplementary Table 1. ICD-9 and ICD-10 codes for identifying myocardial infarction and heart failure.

|  | ICD-9 codes | ICD-10 codes |
| --- | --- | --- |
| Myocardial Infarction | 41000, 41001, 41002, 41010, 41011, 41012, 41020, 41021, 41022, 41030, 41031, 41032, 41040, 41041, 41042, 41050, 41051, 41052, 41080, 41081, 41082, 41090, 41091, 41092 | I21, I219, I230, I231, I232, I233, I234, I235, I236, I238, I210, I2101, I2102, I2109, I211, I2111, I2119, I2121, I2129, I213, I214, I21A1, I21A9, I222 |
| Heart Failure | 4280, 42832, 42822, 42833, 42823, 42830, 42831, 42841, 42842, 42843, 4289, 42840, 4281, 4289 | I5032, I5033, I5022, I5023, I509, I5030, I5031, I5043, I5042, I5041, I5084, I50810, I50814, I50811, I50813, I50812, I5089, I5083, I5021, I5020, I5030, I5031 |

Supplementary Table 2.Associations of ePVS(Hakim formula) with in-hospital death (in logistic analysis model), 180-day death and 1-year death (in cox analysis model)

|  | In-hospital death | | | 180-day death | | | 1-year death | | |
| --- | --- | --- | --- | --- | --- | --- | --- | --- | --- |
|  | OR (95% CI) | P Value | P for trend | HR (95% CI) | P Value | P for trend | HR (95% CI) | P Value | P for trend |
| Model 1 |  |  |  |  |  | <0.001 |  |  | <0.001 |
| Quartile 1 | Reference |  | 0.024 | Reference |  |  | Reference |  |  |
| Quartile 2 | 1.18 (0.88, 1.58) | 0.264 |  | 1.33 (1.05, 1.67) | 0.017 |  | 1.36 (1.09, 1.69) | 0.006 |  |
| Quartile 3 | 1.11 (0.82, 1.49) | 0.497 |  | 1.67 (1.33, 2.09) | <0.001 |  | 1.77 (1.42, 2.19) | <0.001 |  |
| Quartile 4 | 1.44 (1.08, 1.91) | 0.012 |  | 2.17 (1.74, 2.71) | <0.001 |  | 2.35 (1.90, 2.90) | <0.001 |  |
| ePVS | 1.01 (1.00, 1.02) | 0.016 |  | 1.29 (1.20, 1.38) | <0.001 |  | 1.33 (1.24, 1.42) | <0.001 |  |
| Model 2 |  |  | 0.4194 |  |  | <0.001 |  |  | <0.001 |
| Quartile 1 | Reference |  |  | Reference |  |  | Reference |  |  |
| Quartile 2 | 1.04 (0.77, 1.40) | 0.811 |  | 1.09 (0.86, 1.38) | 0.501 |  | 1.11 (0.88, 1.39) | 0.374 |  |
| Quartile 3 | 0.91 (0.67, 1.24) | 0.558 |  | 1.26 (0.99, 1.59) | 0.056 |  | 1.32 (1.06, 1.66) | 0.014 |  |
| Quartile 4 | 1.17 (0.87, 1.57) | 0.296 |  | 1.63 (1.29, 2.05) | <0.001 |  | 1.77 (1.41, 2.21) | <0.001 |  |
| ePVS | 1.00 (0.99, 1.01) | 0.427 |  | 1.18 (1.10, 1.27) | <0.001 |  | 1.21 (1.13, 1.30) | <0.001 |  |
| Model 3 |  |  | 0.1655 |  |  | <0.001 |  |  | <0.001 |
| Quartile 1 | Reference |  |  | Reference |  |  | Reference |  |  |
| Quartile 2 | 1.13 (0.83, 1.54) | 0.428 |  | 1.16 (0.91, 1.48) | 0.240 |  | 1.17 (0.93, 1.48) | 0.175 |  |
| Quartile 3 | 1.00 (0.73, 1.37) | 0.997 |  | 1.37 (1.07, 1.74) | 0.011 |  | 1.44 (1.14, 1.81) | 0.002 |  |
| Quartile 4 | 1.32 (0.97, 1.79) | 0.077 |  | 1.77 (1.39, 2.26) <0.0001 | <0.001 |  | 1.91 (1.52, 2.40) <0.0001 | <0.001 |  |
| ePVS | 1.08 (0.98, 1.19) | 0.141 |  | 1.21 (1.12, 1.31) <0.0001 | <0.001 |  | 1.24 (1.15, 1.34) <0.0001 | <0.001 |  |

Supplementary Figure 1. Generalized Additive Model of ePVS (Hakim formula) for in-hospital mortality prediction


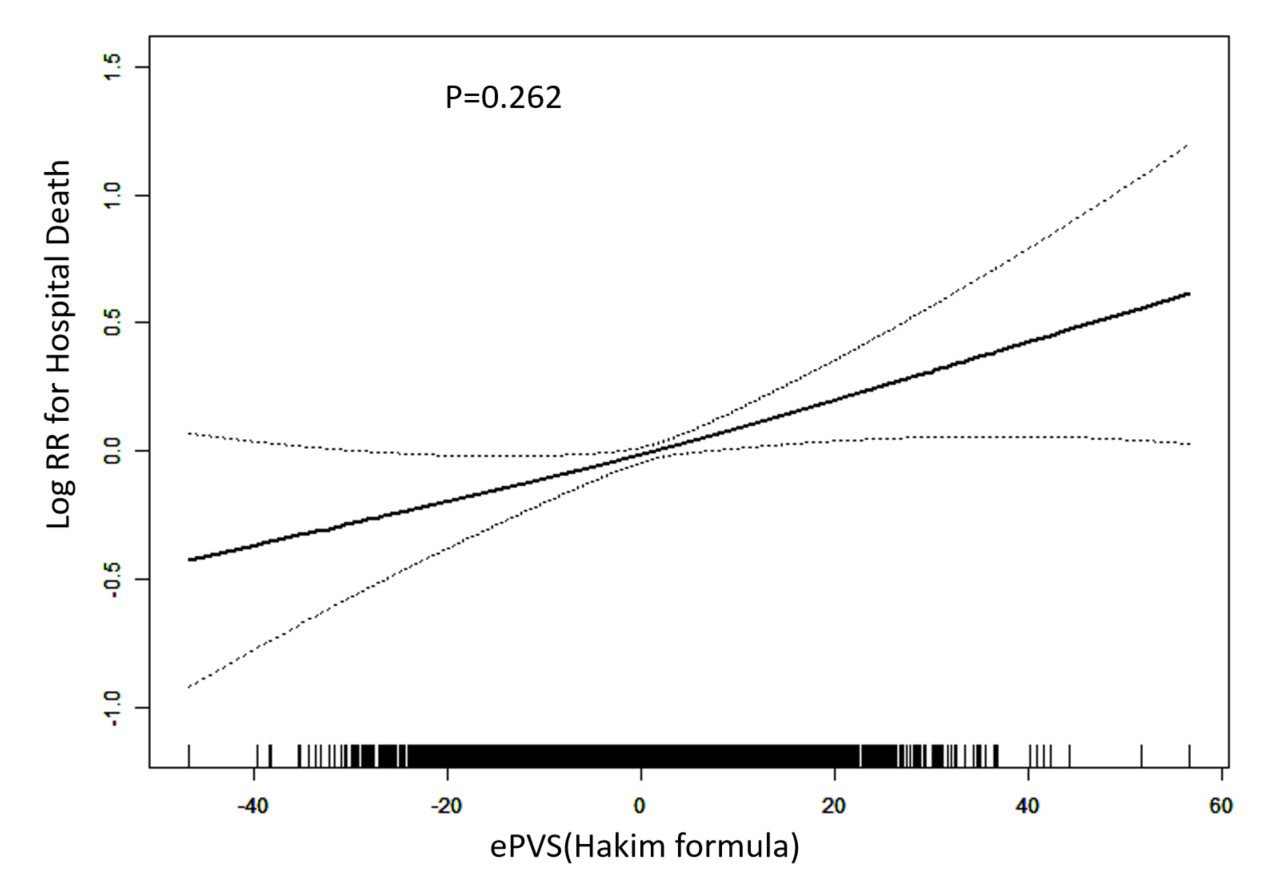


Supplementary Figure 2. Restricted cubic spline (RCS) plots of ePVS (Hakim formula) for predicting 180-day mortality


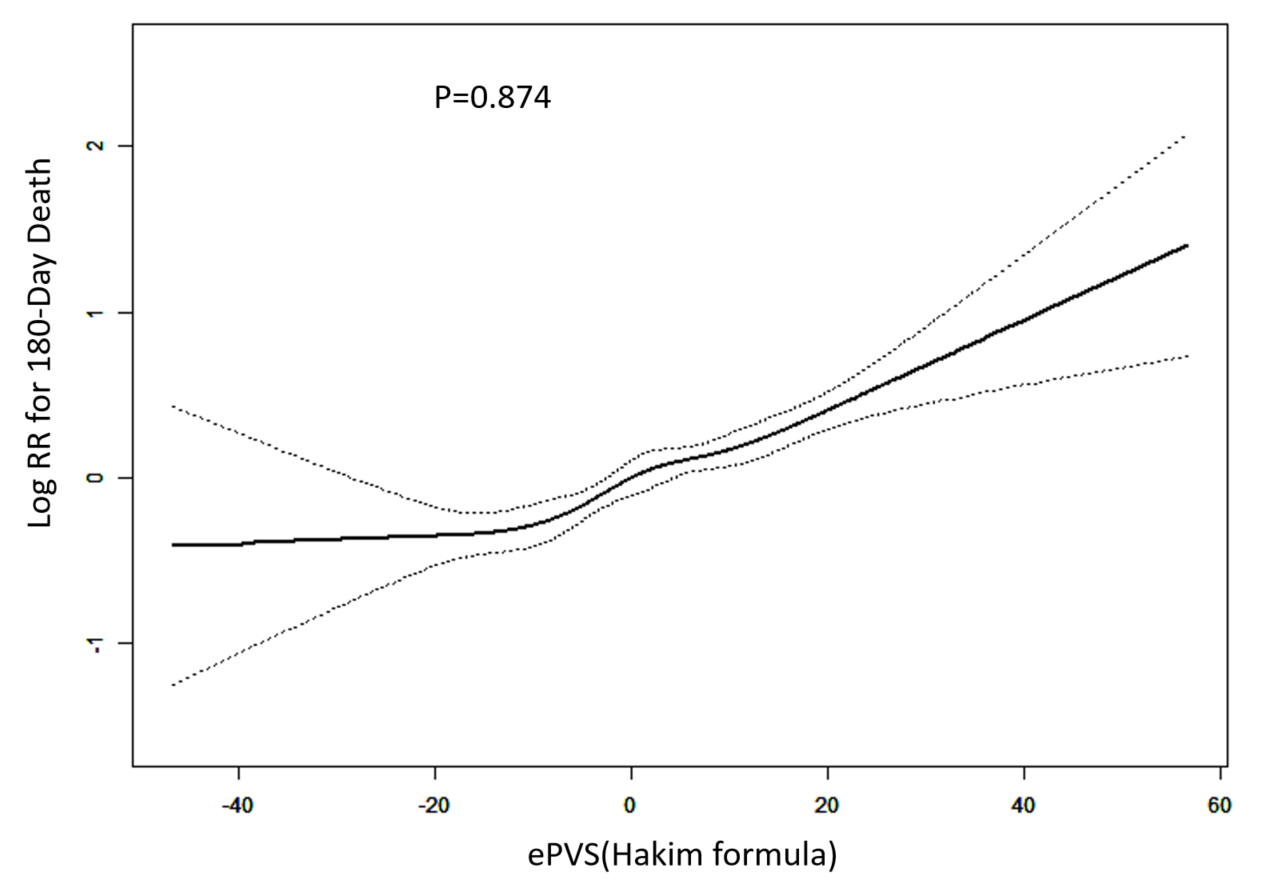


Supplementary Figure 3.RCS plots of ePVS (Hakim formula) for predicting 1-year mortality predictions.


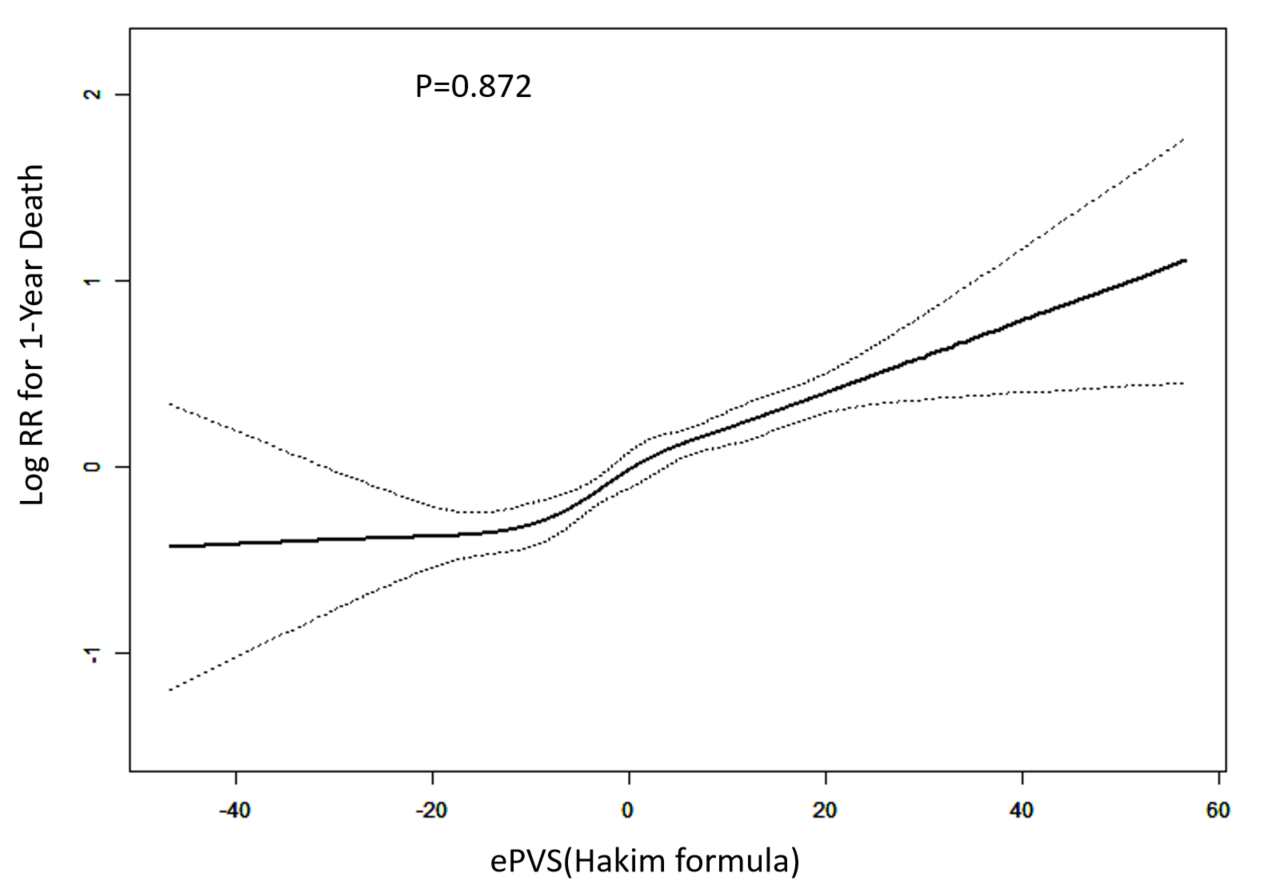


Supplementary Figure 4. Kaplan-Meier analysis of ePVS(Hakim formula) for predicting 180-day mortality


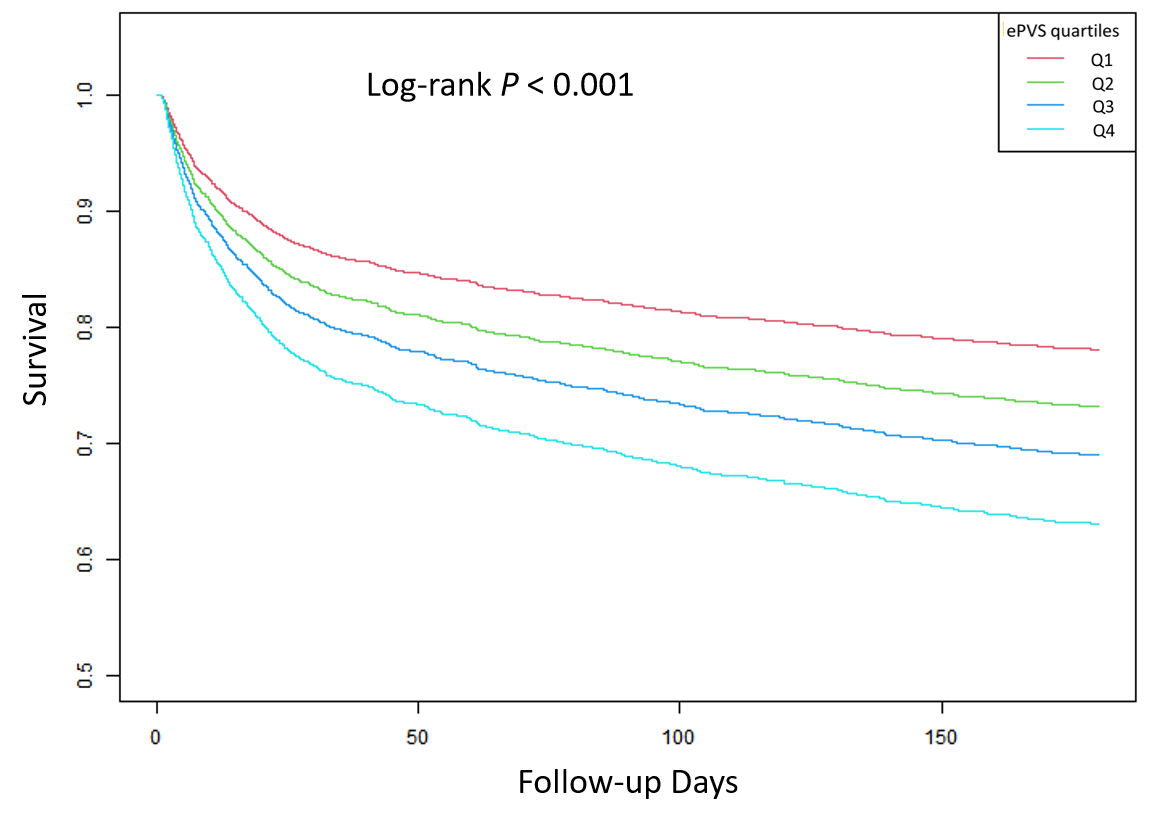


Supplementary Figure 5. Kaplan-Meier analysis of ePVS(Hakim formula) for predicting 1-year mortality predictions.


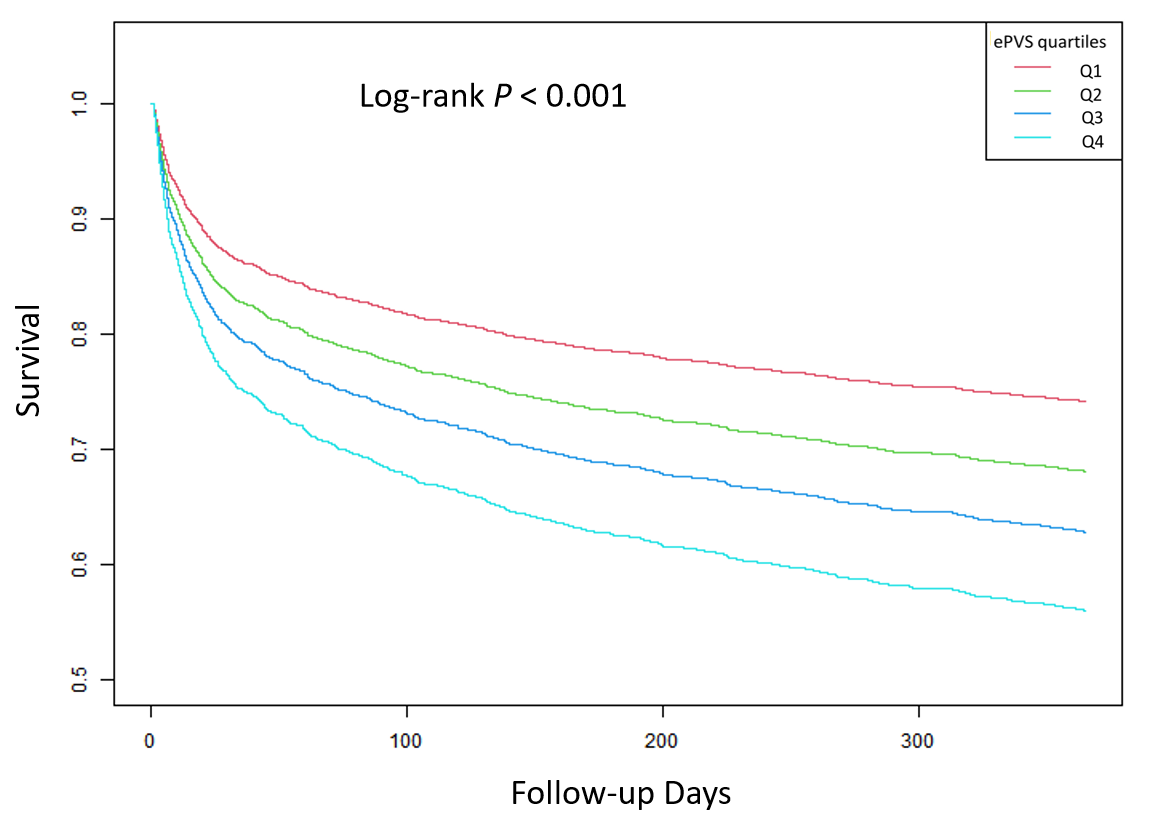

Supplement: Supplementary file 1 [file Datasheet1.docx]
